# The overexpressed functional transient receptor potential channel TRPM2 in oral squamous cell carcinoma

**DOI:** 10.1038/srep38471

**Published:** 2016-12-23

**Authors:** Ling-Yan Zhao, Wan-Lin Xu, Zeng-Qi Xu, Cui Qi, Yang Li, Jie Cheng, Lai-Kui Liu, Yu-Nong Wu, Jun Gao, Jin-Hai Ye

**Affiliations:** 1 Jiangsu Key Laboratory of Oral Diseases and Department of Oral and maxillofacial surgery, Affiliated Hospital of Stomatology, Nanjing Medical University, 136 Hanzhong Road, Nanjing, 210029, China; 2 Shanghai Key Laboratory of Stomatology and Shanghai Research Institute of Stomatology, Department of Oral and Maxillofacial-Head and Neck Oncology, Ninth People’s Hospital, School of Medicine, Shanghai Jiao Tong University, Shanghai, 200011, China; 3 Key Laboratory of Human Functional Genomics of Jiangsu, Department of Neurobiology, Nanjing Medical University, 101 Longmian Road, Nanjing, Jiangsu, 211166, China

## Abstract

TRPM2, one member of the transient receptor potential (TRP) protein super-family, is a Ca^2+^-permeable channel that is activated by oxidative stress and confers susceptibility to cell death. In the human tongue specimens of carcinoma and the tongue carcinoma SCC cell lines, we observed the enhanced expression of TRPM2. By means of the whole-cell electrophysiological recording, the ADPR-induced currents mediated by TRPM2 were recorded in cultured SCC9 cells. Moreover, after H_2_O_2_ treatment for 24 hours, the apoptotic number of SCC9 cells was significantly increased. However, the selectively knocked-down TRPM2 with the small interfering RNA technique inhibited the survival and migration of the SCC9 cancer cells, which was independent of the p53-p21 pathway, since the expression of p21 was enhanced after TRPM2 knockdown. Furthermore, the sub-cellular localization of TRPM2 was remarkably different between cancerous and non-cancerous cells. A significant amount of the TRPM2 proteins were located in the nuclei in cancer cells. All these data suggest that TRPM2 is essential for the survival and migration of SCC cancer cells and may be a potential target for the selective treatment of tongue cancer.

More than 350,000 new cases of head and neck cancer are reported per year^1^, and among them, squamous cell carcinoma (SCC) is the most frequently observed type of oral cancer, which is associated with high morbidity and poor prognosis^2^. An imbalance between cell proliferation and death, tissue invasion and metastasis are exceedingly complex processes, which are the hallmarks of cancer. Therefore, apoptosis induction and the inhibition of cancer invasion and metastasis are currently recognized as the key strategies for inhibiting proliferation of cancer cells. Calcium (Ca^2+^) is an intracellular second messenger which regulates numerous cancer processes ranging from cell proliferation to cell motility^3^. Some of the proteins involved in Ca^2+^ homeostasis are known to be associated with tumor progression. Transient receptor potential (TRP) channels are important modulators of Ca^2+^ homoeostasis which allow calcium into the cells. Aberrant expression of these channels is involved in the development of several cancers including oral squamous carcinoma^4^,^5^.

The TRP channels are initially identified in *Drosophila* melanogaster and named after their role in phototransduction^6^,^7^,^8^. These subfamilies encompass 28 ion channels functioning as diverse cellular sensors. Based on homology and partially on channel function, mammalian TRP channels are classified into six subfamilies: C (canonical), V (vanilloid receptor), M (melastatin), A (ANKTM), P (polycystin), and ML (mucolipin). Different subfamilies mediate a broad range of physiological processes involved in some diseases. The TRPM subfamily of TRP channels are found to be playing important roles in the proliferation and survival of cells. This subfamily was named after the first member, TRPM1 (or melastatin), a putative tumor suppressor protein^9^,^10^. TRPM2 is the second member of the TRPM subfamily to be cloned and expressed in many cell types^11^. Extracellular signals that activate TRPM2 include oxidative stress, TNFα, amyloid β-peptide, and concanavalin A^12^,^13^,^14^,^15^. Stimulation with these extracellular signals results in production of ADP-ribose (ADPR), which activates TRPM2 by being bound to the TRPM2 COOH-terminal NUDT9-H domain, an ADPR hydrolase^16^,^17^. Moreover, intracellular Ca^2+^ can greatly facilitate the activation of TRPM2 channels or the TRPM2 channels^18^,^19^,^20^. The channels of TRPM2 are reported to be temperature-sensitive, and modulated by extracellular pH and zinc^21^,^22^,^23^,^24^. Previous studies have documented functional TRPM2 channels in neurons^12^,^25^,^26^, pancreatic β-cells^21^,^27^,^28^, endothelial cells^29^ and immune cells such as monocytes and lymphocytes^30^,^31^,^32^,^33^,^34^. But the role of TRPM2 in oral cancer is still seldom studied.

More recently, TRPM2 is reported to be activated by irradiation via PARP1 activation and be contributing to irreversible loss of salivary gland function^35^, which suggests that the disordered expression of TRPM2 may be associated with the occurrence of head squamous cell carcinoma (HSCC). The aim of this study is to assess the expression of TRPM2 channels in human HSCC compared with adjacent normal tissue and papilloma tissue, as well as its functional expression in the highly HSCC cancer cell lines (SCC-9). We further evaluated the role of TRPM2 in the regulation of cell apoptosis and migration, and elucidated the probable mechanism.

## Results

### Overexpression of TRPM2 in Human Carcinoma of Tongue Specimens

**Figure 1:**
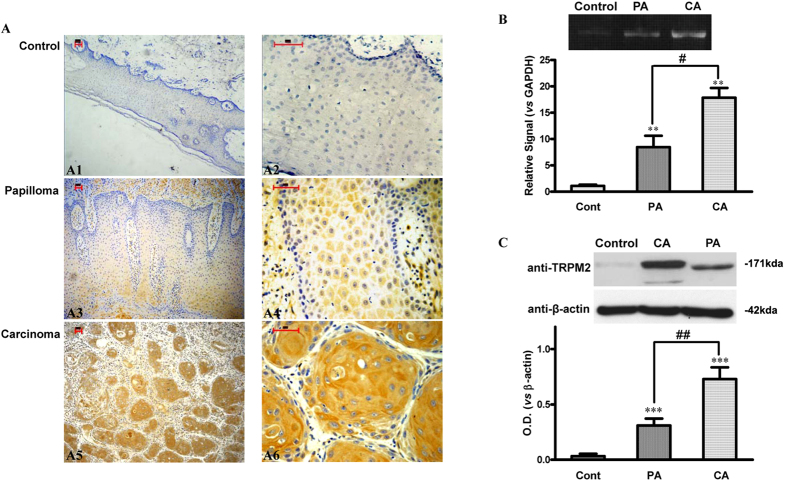
Enhanced Expression of TRPM2 in Human Carcinoma of Tongue Specimens. (**A**) Highly positive immunohistochemical staining of TRPM2 in human carcinoma of tongue specimens (C1, ×40; C2, ×200), negative staining of TRPM2 in non-malignant tongue tissues (A1, ×40; A2, ×200), and weakly TRPM2 positive staining in papilloma of tongue specimens (B1, ×40; B2, ×200). Bar = 50 μm. (**B**) TRPM2 mRNA levels are increased in the tongue specimens of carcinoma. TRPM2 mRNA levels were quantified for real-time PCR and displayed as average ± s.e.m. in the histogram (n = 3 per group). (**C**) Expression of TRPM2 protein levels in the tongue specimens of carcinoma is enhanced compared with that in control and papilloma groups. The tongue specimens from carcinoma, papilloma and control patients (n = 3 per group) are dissected and subjected to western blotting, and optical density is displayed as average ± s.e.m. in the histogram. **p < 0.01, ***p < 0.001 compared with the control group. ^#^*p* < 0.05, ^##^*p* < 0.01 compared with the papilloma group.

We first observed the expression of TRPM2 channels in human normal and cancerous tongue by immunohistochemistry. As shown in Fig. 1A, no obvious staining of TRPM2 was observed in normal tongue tissues (Control, n = 9), while weakly positive TRPM2 staining was observed in 6/12 papilloma of tongue specimens (PA) and negative TRPM2 staining was observed in the rest of papilloma specimens. Through analysis of 23 human tongue carcinoma samples, 22 specimens showed an overexpression of TRPM2, and one weakly positive TRPM2 staining was observed. The specificity of the TRPM2 antibody staining was checked by omitting the primary antibody and blocking the primary antibody with the TRPM2 peptide. To confirm the observation results made on these patients, mRNA from three normal, PA and tumor tissues of each group were extracted and analyzed by semiquantitative PCR (Fig. 1B). In tongue tumor tissues, the mRNA levels of TRPM2 were significantly increased compared with those in the control or PA group. Consistent with it, the high expression levels of TRPM2 protein were also observed in tongue carcinoma samples by Western Blot test compared with the control group (Fig. 1C). All these results suggested that the expression of TRPM2 was elevated in human carcinoma of tongue specimens.

### Overexpression of TRPM2 in Human Tongue Squamous Carcinoma Cell Lines

**Figure 2:**
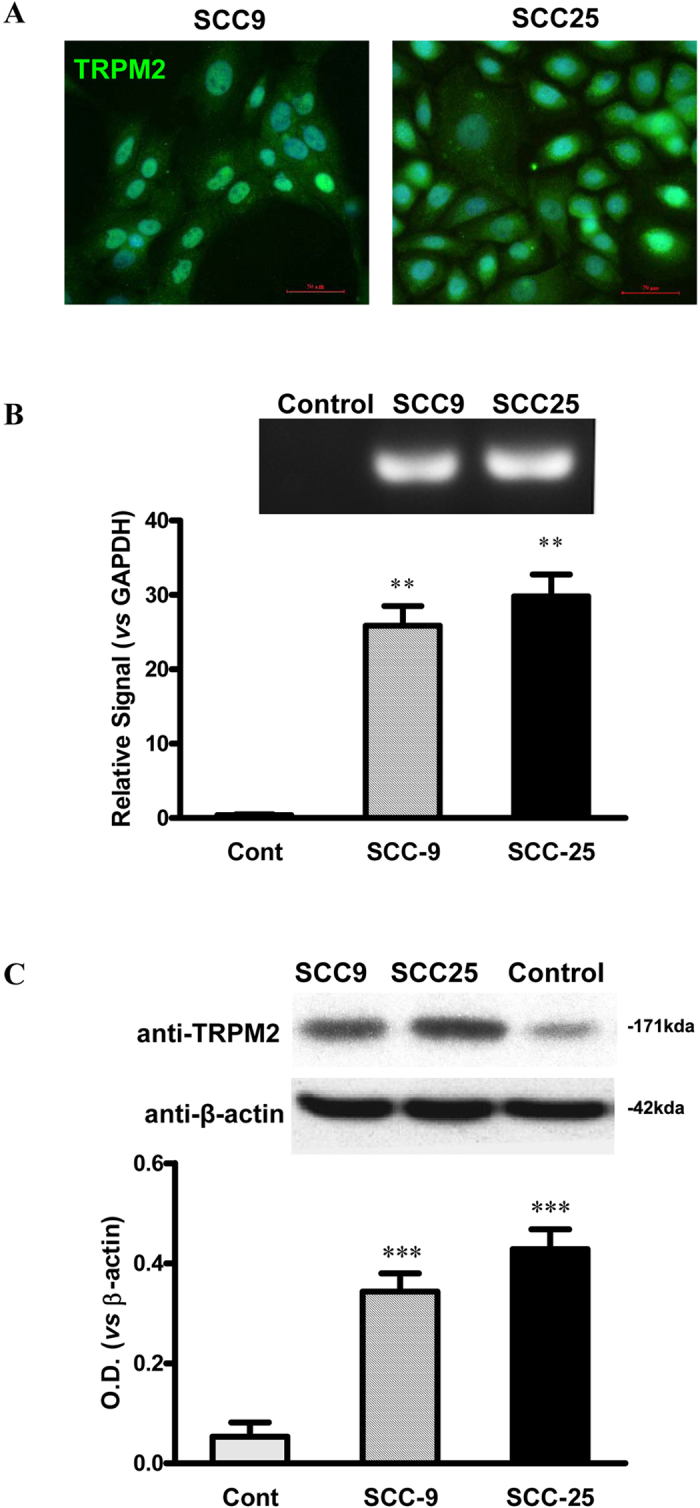
Up-regulated TRPM2 in Carcinoma Cell Lines. (**A**) Positive TRPM2 staining in tongue carcinoma SCC9 and SCC25 cell lines are observed. (**B**) In tongue carcinoma SCC9 and SCC25 cell lines, TRPM2 mRNA levels are enhanced. (**C**) Expression of TRPM2 protein levels in the SCC9 and SCC25 cell lines is increased compared with that of control cells (n = 3 per group). **p < 0.01, ***p < 0.001 compared with the control group.

To investigate the role of TRPM2 in carcinoma, we used the human tongue squamous cell lines, namely SCC-9 and SCC-25 cells, and the non-tumorigenic oral epithelial cell line HIOEC as the control. Consistent with the data from human tongue tumor samples, the positive staining of TRPM2 was observed in SCC-9 and SCC-25 cells (Fig. 2A). Moreover, analysis from Q-PCR and Western-blot also demonstrated the increased levels of TRPM2 mRNA and protein in SCC-9 and SCC-25 cells compared with those in HIOEC cells (Fig. 2B and C). Taken together, TRPM2 was up-regulated in human tongue squamous cell lines.

### Currents Mediated by TRPM2 in Human Tongue Squamous Cell Lines

**Figure 3:**
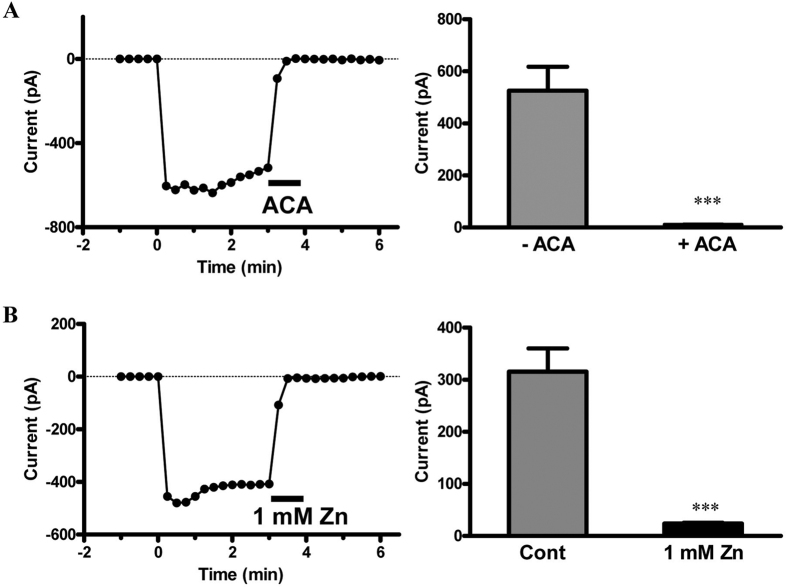
ADPR-induced Currents in Cultured SCC-9 Cells. (**A**) ADPR-induced currents at −80 mV and the I-V relationship curves for TRPM2 channels were recorded using 1-s voltage ramps of −120 mV to 80 mV which were applied every 5 s, in pH 7.3 before and after application of 20 μM ACA. Right panel: Statistical data from the triggered currents by ADPR with or without ACA (n = 18~26 cells). (**B**) Representative recording of ADPR-induced inward currents at −80 mV was blocked by extracellular 1 mM Zn^2+^. Right panel: Statistical data from the triggered current by ADPR with or without 1 mM Zn^2+^ (n = 18~22 cells). ***p < 0.001 compared with the currents before application of 20 μM ACA or 1 mM Zn^2+^.

TRPM2 is a member of the melastatin subfamily of transient receptor potential proteins and forms Ca^2+^ permeable cationic channels gated by intracellular ADPR or structurally related molecules. Thus, the whole cell electrophysiological recordings were made to measure ADPR-induced currents in SCC-9 cells. The currents were evoked by 1-s ramps from −120 to +80 mV every 5 s and the membrane potential was held at −40 mV. ADPR (1 mM) applied via the intracellular solution induced currents of several hundred pA at −80 mV with the typical TRPM2 channel properties such as linearity of I-V curves and strong sensitivity to inhibition by ACA (Fig. 3A) as previously reported. Moreover, recent data showed that Zn^2+^ inactivated the TRPM2 channels and that residues in the outer pore were critical determinants of the inactivation. Consistent with it, we also observed the inhibition effect of 1 mM Zn^2+^ on ADPR-induced currents in SCC-9 cells (Fig. 3B). All these data suggested that TRPM2 channel was functionally expressed in SCC-9 cells.

### Susceptibility of SCC Cells to Death Induced by Oxidative Stress

**Figure 4:**
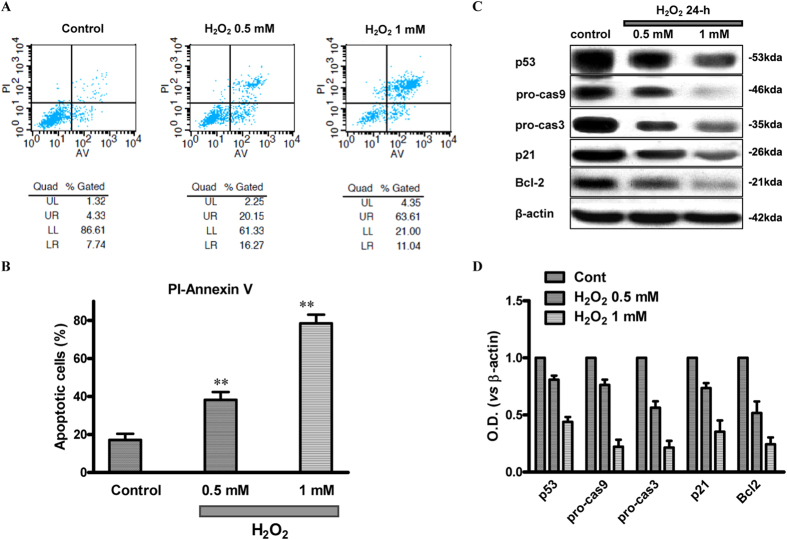
Increased Apoptosis in SCC9 Cells after H_2_O_2_ Treatment. (**A**) The number of apoptotic cells was measured by staining with Annexin V-FITC and PI, and with flow cytometry techniques. The X-axis indicates the intensity of Annexin V-FITC staining and the Y-axis that of PI. Viable cells at the bottom left are FITC^−^PI^−^; early apoptotic cells at the bottom right are FITC^+^PI^−^; cells in late apoptosis at top right are FITC^+^PI^+^; and dead cells at top left are FITC^−^PI^+^. (**B**) Apoptotic cells were elevated after SCC9 cells were treated by 0.5 mM or 1 mM H_2_O_2_ for 24 hours. (control: 13 ± 1%, 0.5 mM H_2_O_2_: 34 ± 2%, and 1 mM H_2_O_2_: 72 ± 2%. n = 3). (**C**) After being treated for 24 hours with different concentrations of H_2_O_2_, expression levels of p21 and p53 in SCC9 cell line were reduced, moreover, caspases-3,-9 and bcl-2 levels also showed a down-regulation trend, all in a dose-dependent manner. (**D**) Quantitative results for the Western blotting of each group (n = 3). **p < 0.01; ***p < 0.001 compared with the control group.

It is well known that TRPM2 channels can be activated by H_2_O_2_. Thus, we observed the effect of different concentrations of H_2_O_2_ on SCC cells. Apoptosis was assessed at 24 hours after H_2_O_2_ treatment by staining with annexin V and PI, followed by flow cytometry. A visual representation of results was shown in Fig. 4A and B. The apoptotic number of SCC9 cells after H_2_O_2_ treatment was significantly increased (ddH_2_O: 13 ± 1%; 0.5 mM H_2_O_2_: 34 ± 2% and 1 mM H_2_O_2_ 72 ± 2%, *p* ＜ 0.001) compared with that of untreated cells, suggesting the involvement of TRPM2 in mediating SCC cells death to oxidative stress. Moreover, it is reported that TRPM2 activation results in caspase-9, -3 cleavage and altered expression of p21 and p53. Thus we further investigated the expression of these proteins in SCC9 cells treated with different concentrations of H_2_O_2_ for 24 hours. The immunoblotting results showed that the expression levels of Bcl-2, pro-caspases-3 and -9 were reduced in a dose-dependent manner in SCC9 cells treated by H_2_O_2_ for 24 hours (Fig. 4C and D). We also examined the alteration of p53 and p21, the tumor-associated factors. As shown in Fig 4C and D, the expression of p53 in SCC cells was gradually reduced after TRPM2 was activated by different concentrations of H_2_O_2_. Moreover, the levels of p21 also showed a down-regulation after H_2_O_2_ treatment for 24 hours in SCC9 cells. All data indicated that TRPM2 was activated by oxidative stress, leading to activation of caspases-3 and -9 and changing of the expression of the related tumor-associated factors, and finally promoting the apoptosis in cultured SCC9 cells.

### Deletion of TRPM2 Inhibits the Migratory Abilities of SCC-9 Cells and Induces Cell Death in SCC-9 Cells

**Figure 5:**
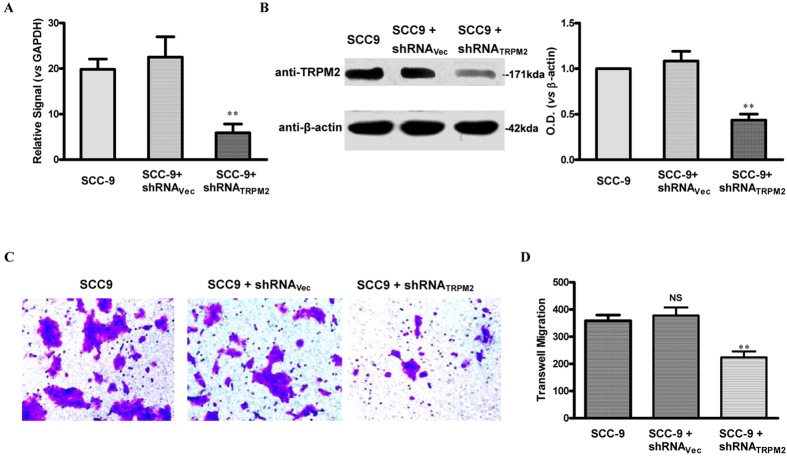
Effect of Down-regulation of Endogenous TRPM2 on the Migration of the SCC9 Cells. (**A**) Real-time PCR revealed that TRPM2 mRNA in group shRNA_TRPM2_ was significantly decreased compared with that of the control and sham groups. TRPM2 mRNA levels were decreased by about 65.6% in SCC9 cells, with no significant difference of TRPM2 mRNA levels found between control and shRNA_Vec_ group (*p* > 0.05, n = 3). (**B**) Western blotting revealed transfection of cells with TRPM2 shRNA for 48 hours significantly suppressed endogenous TRPM2 protein expression in SCC9 cells, with no significant difference between control and shRNA_Vec_ group (n = 3). (**C**) Transwell assay showed the migrated and stained cells at 24 hours after being transfected with or without shRNA_TRPM2_ plasmid. (**D**) Quantitative results for the transmembrane ability of each group of cells (n = 3). **p < 0.01 represents cell migration in shRNA_TRPM2_ transfected group compared with the shRNA_Vec_ blank group at 24 h transfection.

**Figure 6:**
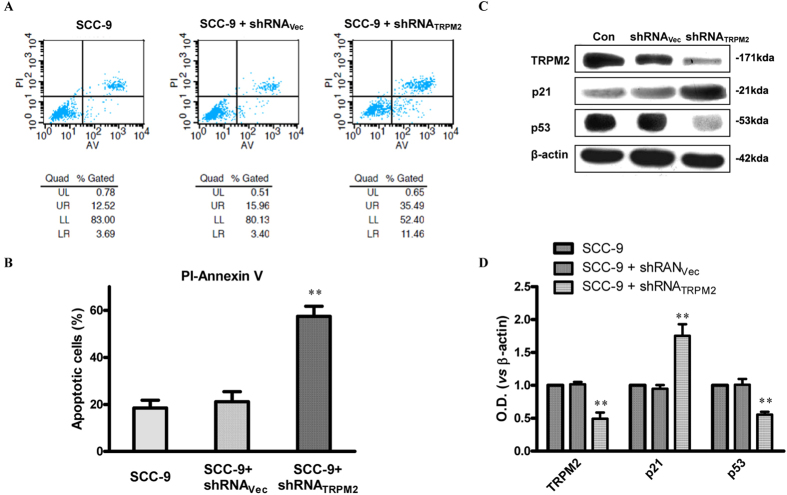
Selectively Down-regulation of TRPM2 in SCC9 Cell Lines Promoted Apoptosis of Cell and Altered the Expression of Related Protein. (**A**) The number of apoptotic cells was measured after SCC9 cells were transfected with shRNA_Vec_ or shRNA_TRPM2_ for 48 hours by staining with Annexin V-FITC and PI and with flow cytometry techniques. (**B**) Apoptotic cells were increased in SCC9 cells 48 hours after being transfected with shRNA_TRPM2_. (control: 18 ± 1%; shRNA_Vec_: 19 ± 1%; and shRNA_TRPM2_: 57 ± 2%. n = 3). (**C**) Western blotting revealed that transfection of SCC9 cells with shRNA_TRPM2_ for 48 hours significantly suppressed the expression of tumor-related protein p53, but increased the level of p21 expression. (**D**) Quantitative results for the Western blotting of each group (n = 3). **p < 0.01 compared with the control group.

To further explore the role of TRPM2 in tongue carcinoma, we inhibited the expression of TRPM2 by transfection with shRNA_TRPM2_ in SCC-9 cells. As shown in Fig. 5A and B, data from Q-PCR and western-blot indicated that the mRNA and protein levels of TRPM2 in SCC-9 cells transfected with shRNA_TRPM2_ for 48 hours were significantly reduced compared with those in SCC-9 cells transfected with or without vector. Next, *in vitro* migration assay was performed (Fig. 5C and D). We found that the dampened expression of TRPM2 could significantly down-regulate the migratory abilities of the SCC-9 cells. Then, we investigated the effect of TRPM2 knockdown on cell apoptosis with flow cytometry techniques. The number of apoptotic cells was measured by double staining with Annexin V-FITC and PI. In control or SCC9 cells transfected with vector, apoptotic cells were 18 ± 1% and 19 ± 1% respectively, while it was significantly increased to 57 ± 2% in SCC-9 cells transfected with shRNA_TRPM2_ (Fig. 6A and B). Consistent with it, the expression of p53 was significantly reduced while that of p21 was enhanced after knockdown of TRPM2 in SCC-9 cells (Fig. 6C and D). All these data suggested that overexpressed TRPM2 tongue carcinoma might be involved in the survival and migration of the cancer cell.

### Subcellular Distribution of TRPM2 is Changed in Cancer Samples

**Figure 7:**
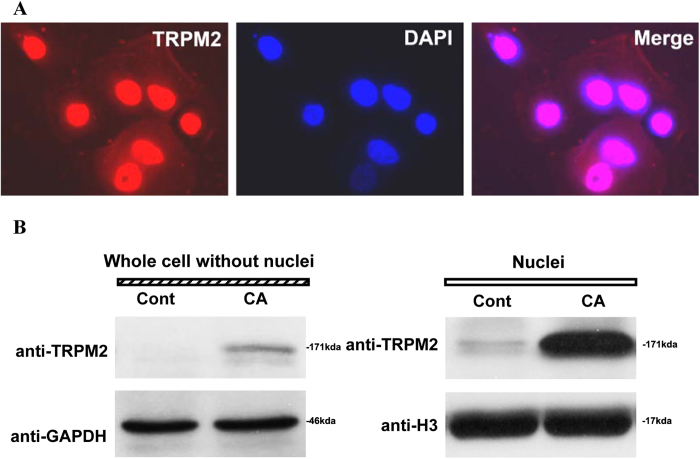
Expressed TRPM2 in the Nuclei is Increased in the SCC9 Cells and the Tongue Specimens of Carcinoma. (**A**) Respective TRPM2 nuclei staining in SCC9 cell lines were observed. Red: TRPM2; Blue: DAPi. (**B**) In the samples from the human tongue carcinoma, TPRM2 was mainly expressed in the nuclei and a little was expressed in the remainders (whole cell preparation excluding nuclei), while no expression of TRPM2 was observed in control tissues.

The above data showed that either activating TRPM2 by H_2_O_2_ or inhibiting TRPM2 by shRNA induced the increased cell apoptosis. Zeng X *et al*. reported that the subcellular localization of TRPM2 was different between prostate cancer and non-cancerous cells, which may contribute to the different functions^36^. Thus we also observed the localization of TRPM2 in tongue carcinoma. As shown in Fig. 7A, the positive TRPM2 staining in nuclei was significantly higher than that in plasmas of SCC9 cells. To confirm the predominant expression of TRPM2 in the nuclei of SCC cells, nuclear proteins from the human tongue carcinoma samples were extracted by western blot analysis. As shown in Fig. 7B, expression of TRPM2 protein was detected in the nuclei of the human tongue carcinoma samples. In contrast, TRPM2 was absent in the nuclei of normal human tongue samples. These data confirmed that TRPM2 was expressed in the nuclei of the human tongue cancer cells. Moreover, we also observed the TRPM2 expression in the cytoplasm preparations in the human tongue carcinoma samples, but not in normal samples.

## Discussion

The present study demonstrates that: 1) TRPM2 channel is highly expressed in SCC cells and tissues, with little or no detectable levels in normal tongue epithelial tissues; 2) TRPM2 is functional in HSCC cell lines; 3) Activation of TRPM2 by H_2_O_2_ increases apoptosis of SCC cells through the p21 and p53 pathway; 4) Inhibition of TRPM2 expression by shRNA_TRPM2_ also increases the apoptosis of SCC cells and reduces the migration of SCC cells; 5) The expression of TRPM2 in nuclei is enhanced in SCC cells. Taken together, our findings suggest that TRPM2 is involved in the regulation of migration and survival of HSCC cells, and the difference in TRPM2 location (membrane or nuclei) may contribute to the different function of TRMP2.

### TRPM2 and Oxidative Stress

It has long been known that ion channels are greatly important for a large variety of physiologic functions including electrical signaling, gene expression, cell volume regulation, hormone secretion and so on. In contrast to these beneficial effects, a lot of studies show that ion channels are also associated with several diseases including cancer^37^,^38^. This relationship has been demonstrated mainly in K^+^ channels. Several other ion channels seem to be associated with cell proliferation including Na^+^, Cl^−^ channels, Ca^2+^ channels etc. Transient receptor potential (TRP) channels are cation channels associated with cancer^4^. For example, the TRPM1 expression level is inversely correlated with melanoma aggressiveness and metastatic potential, suggesting that it functions as a tumor suppressor^9^,^10^. To date, other TRPM members including TRPM2, 4, 5, 7 and 8 have also been proven to be associated with the proliferation of and survival of cells. Among TRP channels, TRPM2 is expressed in many non-cancerous cells, such as brain and peripheral blood cells. Large amounts of studies show that TRPM2 is associated with many types of diseases. The ability of H_2_O_2_ to activate TRPM2 channels has attracted interest in this channel as a potential mechanism for pathogenic processes characterized by increased oxidative microenvironment, including carcinogenesis, inflammation, ischemia-reperfusion injury, neurodegenerative disorders and diabetes^11^,^39^,^40^. The first evidence indicating that TRPM2 activity is modulated by oxidative stress was reported in rat pancreas cells. Then a lot of evidence demonstrated the connection between TRPM2 activity and oxidative stress^41^,^42^,^43^. Moreover, cells expressing endogenous or transfected with TRPM2 exposed to micro molar H_2_O_2_ levels showed an increased TRPM2 current^14^. Reactive oxygen species (ROS) are able to modify the function of TRPM2 and produce functional changes, which become imbalanced in cellular homeostasis. Moreover, the increased reactive oxygen species (ROS) by oxidative stress can enhance ADPR production, which activates full-length TRPM2 (TRPM2-L). In fact, in the present study, we also recorded the ADPR-induced currents in cultured SCC-9 cells (Fig. 3) and observed the increased apoptosis after SCC-9 cells were treated with 0.5 mM or 1 mM H_2_O_2_ for 24 hours (Fig. 4). The function of TRPM2 in these cells is to serve as a mediator of oxidative-stress-induced calcium influx (i.e., the channel is at plasma membrane) and thus apoptosis in those tissues, so inhibition of TRPM2 may be beneficial in terms of continued survival. However, in our study, we observed the increased apoptosis and the inhibited migration of SCC9 cells after knockdown of TRPM2 with shRNA _TRPM2_ (Figs 5 and 6).

### Different Transcripts and Locations Contribute to Different Functions of TRPM2

Two TRPM2 transcripts, namely TRPM2-S and TRPM2-L, have been reported^44^,^45^. TRPM2-S is a physiological TRPM2 splice variant lacking four of the six predicted COOH terminal transmembrane domains and the putative Ca^2+^ pore. Both transcripts seem to be up-regulated in cancer cells with little or no detectable levels in control cells. Though coexpression of TRPM2-S does not change the subcellular localization of TRPM2-L, the expression of TRPM2-S inhibits susceptibility to cell death and onset of apoptosis induced by H_2_O_2_ in cells expressing TRPM2-L^44^. Recent data from Chen SJ *et al*. suggest that overexpression of TRPM2-S results in increased proliferation through phosphatidylinositol 3-kinase/Akt and ERK pathways in the neuroblastoma SH-SY5Y cell lines, while overexpression of TRPM2-L confers protection against oxidative stress-induced cell death through FOXO3a and SOD^46^,^47^. All these data suggest the functional difference of TRPM2 isoform in cells.

Previous studies have shown that TRPM2 channels are expressed primarily in the plasma membrane. Data from Zeng X. *et al*.^36^ first showed that the subcellular localization of TRPM2 was also remarkably different between cancerous and non-cancerous cells. In BPH-1 (benign), TRPM2 protein was homogenously located near the plasma membrane and in the cytoplasm, whereas in the cancerous cells (PC-3 and DU-145), a significant amount of the clustered TRPM2 protein was located in the nuclei. Moreover, the selectively knocked down TRPM2 inhibited the growth of prostate cancer cells but not of non-cancerous cells^36^. Recent data from Hopkins MM *et al*. demonstrated that TRPM2 was present in the nuclei of MCF-7 and MDA-MB-231 human breast adenocarcinoma cells, and its pharmacologic inhibition or RNAi silencing caused the decreased cell proliferation and the 4-fold increases in DNA damage levels, suggesting a novel effect of TRPM2, where it functions to minimize DNA damage and thus may have a role in the protection of genomic DNA in breast cancer cells^48^. All of these data suggested that, during the change process from a normal cell toward cancer, a series of genetic alterations take place, which may affect the expression and location of ion channels or induce changes in channel property/activity. The abnormal ion channel property/activity is then able to promote the growth and proliferation of tumor. The L-type Ca^2+^ channel has been reported to be proteolytically cleaved to form a C-terminal fragment, which is translocated into the nuclear and changes the gene transcription^49^. In the present data, we also observed the nuclear location of TRPM2 in the tongue cancerous cells (Fig. 7). To date, TRPM2 is reported to be mainly expressed in the plasma membrane of several cell types, including microglial cells^50^,^51^, neutrophil granulocytes^33^,^52^, immune cells^30^,^53^, insulin-secreting cells^21^,^54^, neurons^25^ and arterial endothelial cells. In non-cancerous cells, the membrane TRPM2 was responded to oxidative stress and mediated Na^+^ and Ca^2+^ influx. In the tongue cancer cells, a number of TRPM2 was observed in the nuclei, though the membrane TRPM2 still performed its function since ADPR-induced current was recorded in SCC-9 cell (Fig. 3). As result, the role of membrane TRPM2 was weakened and the nuclei TRPM2 was pivotal for the proliferation and migration of the SCC cells since the knockdown of TRPM2 inhibited the migratory abilities and enhanced the susceptibility of SCC cells. And this function was independent of the p53-p21 pathway since the expression of p21 was up-regulated after the deletion of TRPM2 (Fig. 6). Moreover, some of TRPM family proteins such as TRPM8 were observed in the endoplasmic reticulum of the cancer cells, and they played a essential role in cancer cell survival^55^,^56^,^57^. Whether TRPM2 also functions as an endoplasmic reticulum channel in cells is worthy of further investigation.

Taken together, our data suggest that the function of the plasma TRPM2 is different from that of the nuclei TRPM2. During carcinogenesis, we propose that TRM2 should act in a biphasic manner as a tumor attenuator and an enhancer of the malignant phenotype. High levels of plasma membrane TRPM2 may be protective against the early stages of neoplastic growth. However, the lack of plasma membrane TRPM2 and an elevated level of nuclei TRPM2 during the advanced stages of cancer may increase susceptibility to tumorigenesis.

## Materials and Methods

### Cell Culture

Human tongue squamous cell carcinoma cell lines, including SCC9 and SCC25, were purchased from ATCC (Manassas, VA, USA). For comparison purposes, human lingual dorsal epithelial cells routinely grown as primary culture in our laboratory and kept in DMEM media containing 10% fetal bovine serum were used as controls in these studies. All cells were cultured in a humidified incubator with 5% CO_2_ and 95% air at 37 °C.

### Patients and tissue specimens

In all 81 cases with primary human tongue SCC or PA, who received surgical treatment during January 2005-December 2014 at the department of oral and maxillofacial surgery, Nanjing Medical University, were enrolled in this study. None of the patients received any prior treatment for SCC. Information about clinical, pathological and follow-up data (follow-up status for 70 patients) were analyzed. All surgically removed tissues were routinely processed, and a single representative block of cancer or PA tissue was selected and used for the immunohistochemistry. Nineteen samples of normal tongue tissues were obtained from other non-cancer surgeries as their counterparts during the same period. All the patients were provided the written informed consents in compliance with the code of ethics of the World Medical Association at the time of surgery for the donation of their tissues for this research. All these tissues were obtained with the consent of the patients for use of the tissue samples for research purposes. The using human tissue in the present study was approved by the Human Ethic Committee of the Nanjing Medical University. The procedures were performed in accordance with the guidelines.

### Immunohistochemistry

Immunocytochemical analysis of recovered implants was performed with the streptavidin-biotin complex method according to the protocol recommended by the manufacturer. In brief, tissue sections (5 µm) from representative paraffin blocks were deparaffinized in xylene and rehydrated through graded ethanol solutions. Endogenous peroxidases were blocked with 3% hydrogen peroxide. With respect to the antigen retrieval, the sections were processed by the conventional microwave heating in 0.01 M sodium citrate retrieval buffer (0.01 M sodium citrate and 0.01 M citric acid, pH 6.0) for 5 minutes. Then, the sections were blocked by 10% normal swine serum for 20 minutes and incubated with primary antibodies (TRPM2, 1:1000) at 4 °C overnight. Incubation with PBS instead of primary antibodies served as the negative controls. Finally, the sections were incubated with secondary antibodies at room temperature for 45 minutes. The reaction products were developed by 3, 3′-diaminobenzidine solution with hydrogen peroxide and counterstained with hematoxylin.

### Western Blot Analysis

Samples from normal or tumor tissues, SCC9 and SCC25 monolayers were washed twice with 1× phosphate-buffered saline, lysed in RIPA lysis buffer (Beyotime, China) containing 1 mM phenylmethylsulfonyl fluoride (PMSF), and then cell debris was eliminated by centrifugation at 12,000 rpm for 10 minutes. The nuclear protein of the carcinoma tissue and normal specimen were extracted according to the protocol of the nuclear protein extraction kit (KeyGEN BioTECH, China). Protein concentrations were determined by Bradford assay. With respect to western blot analysis, an equal amount of protein (40 μg) from each cell extraction was separated in SDS–PAGE gels. The proteins were transferred electrophoretically to a 0.4 µm PVDF membrane (Millipore Co. Bedford, MA, USA) at 300 mA for 2 hours. Non-fat dry milk (5%) in TBS containing 0.1% Tween 20 (TBS-T) was used as a blocking agent at room temperature with constant agitation for 2 hours. The membrane was then treated with polyclonal primary antibody against TRPM2 (1:1000; Abcam, Cambridge, MA), pro-caspase3 (1:1000; Bioworlde, USA), pro-caspase9 (1:1000; Bioworlde, USA), p53 (1:1000; Bioworlde, USA), p21 (1:1000; Bioworlde, USA), bcl-2 (1:1000; Bioworlde, USA) and monoclonal antibody against β-actin (1:300; Boster Inc., Wuhan, China) at 4 °C overnight. It was rinsed briefly with TBS-T three times, and incubated with HRP-conjugated secondary antibody (dilution 1:5000 in TBS-T) at room temperature with constant agitation for 1 hour. The membrane was developed with the ECL western blot detection reagent kit (Bio-Rad Laboratories, Hercules, CA, USA) and exposed to Kodak X-ray films. β-actin served as an internal control in these experiments.

### TRPM2 siRNA Synthesis and Transfection

A specific short hairpin RNA (shRNA) against human TRPM2 was purchased from Santa Cruz (USA). One day before transfection, 1.5 × 10^5^ SCC cells were placed in 2 ml growth medium without antibiotics per well in a 6-well plate (Costar). On the 2^nd^ day, 10 μL shRNA vector (100 ng/μL) or 10 μL Lipofectamine ^TM^ 2000 (Invitrogen,, Carlsbad, CA, USA) was diluted in 50 μL Opti-MEM medium without serum (GIBCO, USA), and gently mixed and incubated at room temperature for 5 minutes. After that, the diluted shRNA vector was combined with the diluted Lipofectamine ^TM^ 2000, then gently mixed and incubated at room temperature for 20 minutes. At the same time, the cells in 6-well plate were washed twice with medium without serum, then added by 2 ml medium without serum in each well. 60 μL of the shRNA- Lipofectamine^TM^ 2000 complexes was added to each well and gently mixed by rocking the plate back and forth. The cells were cultured in a humidified incubator with 5% CO_2_ and 95% air at 37 °C for 6 hours, then the medium was changed to the one containing serum. After the cells were incubated in the same circumstance for another 72 hours, we collected the transfected cells for assays.

### RNA Extraction and Detection by semiquantitative PCR or Real-time PCR

Total RNA was isolated from cultured cells by using TRIzol Reagent (Invitrogen,, Carlsbad, CA, USA) according to the manufacturer’s protocol. Isolated RNA precipitates were completely dissolved in diethypyrocarbonate (DEPC) treated water (Ambion Inc., Austin, USA) and reversely transcribed with ImpromII^TM^ Reverse Transcription System kit (Promega, USA). The isolated RNA precipitates were completely dissolved in diethypyrocarbonate (DEPC) treated water (Ambion Inc., Austin, USA) and reversely transcribed with ImpromIITM Reverse Transcription System kit (Promega, USA).

The semiquantitative PCR reaction was performed using Ex Taq DNA polymerase (Promega, USA) for TRPM2 gene (Invitrogen, USA). The housekeeping gene glyceraldehydes-3-phosphate dehydrogenase (GAPDH) was co-amplified, using specific primers for the human gene, as a constitutive control. The real-time RT-PCR was performed by using iTaq^TM^ SYBR Green Supermix with ROX kit (Bio-Rad Laboratories, Hercules, CA, USA) and ABI 7300 real-time PCR system (Perkin-Elmer, Applied biosystems). Real-time RT-PCR reaction conditions were as follows: 95 °C for 2 minutes and 30 seconds, followed by 40 cycles of 95 °C for 10 seconds, and 55 °C for 40 seconds.

The primers of TRPM2 were forward: 5′-TGGTGCGAGTGTGCCATCTAC-3′ and reverse: 5′-CGCACTCGTCAGGGTCATAGAA-3′. The primers of GAPDH were forward: 5′-CTCCATCCTGGCCTCGCTGT-3′ and reverse: 5′-GCTGTCACCTTCACCGTTCC-3′. The primers of β-actin were forward: 5′-TGGCACCCAGCACAATGAA-3′ and reverse: 5′-CTAAGTCATAGTCCGCCTAGAAGCA-3′. Resulting products were visualized on a 1.5% agarose gel with ethidium bromide staining. The intensity of the resulting bands was quantified under UV light and normalized with reference to GAPDH mRNA.

### Flow Cytometric Analysis

With respect to flow cytometric analysis, the cells were firstly synchronized with serum-free medium for 24 hours. Next, the cells were treated with mitomycin for another 24 hours. Then the prepared cells were collected and stained with PE Annexin V Apoptosis Detection Kit I (BD Pharmingen, USA) for 15 minutes according to the manufacturer’s protocol. Compared with each untreated group, the rate of apoptosis cells was counted and analyzed.

### *In Vitro* Cell Migration Assay

The cell migration assay was assessed by using Transwell filters with 6.5-mm diameters and 8-μm pore sizes (Costar, USA). The cells (2.5 × 10^5^) were re-suspended with 100 μl serum-free medium inoculated in the upper chamber, while 500 μl medium containing 10% FBS was placed in the lower chamber. The plates were placed in the incubator for 24 hours. The chambers were fixed with 4% PFA and stained with 0.1% crystal violet (Beyotime, China) for 10 minutes. The migratory cells were counted as those present on the lower surface of the upper chamber. Images of at least ten random fields per chamber were photographed and counted.

### Statistics

The quantitative results were expressed as mean ± SE. Independent samples *t*-test and Chi-square test were performed with SPSS-Windows v.12.0 software. *P*-values less than 0.05 were considered as statistically significant.

## Additional Information

**How to cite this article**: Zhao, L.-Y. *et al*. The overexpressed functional transient receptor potential channel TRPM2 in oral squamous cell carcinoma. *Sci. Rep.* **6**, 38471; doi: 10.1038/srep38471 (2016).

**Publisher's note:** Springer Nature remains neutral with regard to jurisdictional claims in published maps and institutional affiliations.
